# Survival and Clinical Outcomes after Unconstrained Total Knee Arthroplasty for Tibial Plateau Fractures–A Retrospective Study with Minimum 4-Year Follow-Up

**DOI:** 10.3390/jcm12237303

**Published:** 2023-11-25

**Authors:** Philip-C. Nolte, Kim Schlentrich, Philipp Raisch, Matthias K. Jung, Paul A. Grützner, Oliver Bischel

**Affiliations:** Department for Trauma and Orthopaedic Surgery, BG Klinik Ludwigshafen, Ludwig-Guttmann-Strasse 13, 67071 Ludwigshafen, Germanyk.schlentrich@web.de (K.S.);

**Keywords:** arthroplasty, complications, knee replacement, orthopaedics, revision surgery, survival, TKA

## Abstract

This study investigated survival, complications, revisions, and patient-reported outcomes (PROs) for unconstrained total knee arthroplasty (TKA) in posttraumatic osteoarthritis (PTO) caused by intraarticular tibial plateau fractures with minimum four years follow-up. Forty-nine patients (71.4% male; 58.7 years) were included. Kaplan–Meier analysis was performed with failure defined as TKA removal. Patients without failure underwent pre- and postoperative evaluation (range of motion (ROM), Oxford Knee Score (OKS), Knee Society Score (KSS), anatomical femorotibial angle (aFTA), proximal tibial slope (PTS)) and Short Form-12 (SF-12) Physical (PCS) and Mental Component Summary (MCS) assessment at final follow-up. Fifteen (30.6%) patients had a complication, and eight (16.3%) patients underwent prosthesis removal at median 2.5 years. Cumulative survival rate of TKA was 79.6% at 20 years. A total of 32 patients with a mean follow-up of 11.8 years underwent further analyses. ROM (*p =* 0.028), aFTA (*p* = 0.044), pPS (*p* = 0.009), OKS (*p* < 0.001) and KSS (*p* < 0.001) improved significantly. SF-12 PCS was 42.3 and MCS was 54.4 at final follow-up. In general, one third of patients suffer a complication, and one in six patients has their prosthesis removed after TKA for PTO due to tibial plateau fractures. In patients who do not fail, TKA significantly improves clinical and radiographic outcomes at long-term follow-up.

## 1. Introduction

More than 300 million people worldwide are affected by osteoarthritis, which is one of the leading causes for pain, disability and socioeconomic costs [1,2]. Of those, approximately 10–12% have posttraumatic osteoarthritis (PTO) that can arise from intra- or extraarticular fractures as well as from ligamentous injuries [3,4,5]. Following such injuries, up to 48% of patients develop PTO within 10 years after injury [3,4,6]. Major risk factors for PTO of the knee include direct intra-articular injury, insufficient (but also sufficient) reduction in or fixation of fractures, residual malalignment with abnormal loading of the articular surface, instability secondary to ligament injuries, young age, and pre-existing joint degeneration [7,8,9].

In PTO, initial treatment is nonoperative and includes activity modification, non-steroidal anti-inflammatory drugs, corticosteroids, ambulatory assist devices, physical therapy and orthoses [8,10]. Ultimately, PTO progresses and nonoperative treatment may fail. For end-stage PTO of the knee, total knee arthroplasty (TKA) is a common procedure that has been shown to alleviate pain and restore function [10,11].

Whereas some authors have demonstrated satisfactory functional outcomes for TKA in PTO similar to those performed for primary osteoarthritis (OA) [10,12,13], others have found inferior patient-reported outcome measures (PROs) and revision-free survival [11,14,15]. Less controversy exists regarding the higher rate of complications for TKA following PTO [10,11,12,14,15,16,17,18]. Among the reasons for the higher complication rate is that in patients with PTO, arthroplasty is technically more demanding due to posttraumatic bone defects, remaining hardware, malalignment, scar tissue, limited range of motion and compromised soft tissue, thus requiring more time in the operating room [14]. One of the most common complications are periprosthetic joint infections (PJIs) with catastrophic consequences for the patient [14,17,19].

Whereas TKA in primary OA has been studied extensively, there is a paucity of literature investigating TKA for PTO. Of the studies available, many include multiple causes for PTO such as fractures of the distal femur, tibial plateau, and patella as well as ligamentous injuries about the knee [15,17,19]. However, outcomes of TKA may differ substantially depending on the type of injury for which the procedure was performed [20].

Therefore, the aim of this study was to investigate survival, complications, and revision surgery as well as patient-reported outcomes in patients who underwent TKA for PTO caused by intraarticular fractures of the tibial plateau. We hypothesized that there would be a high rate of complications and revisions, but PROs would be satisfactory.

## 2. Patients and Methods

In this retrospective, single-center study, patients that underwent TKA for PTO following intraarticular fractures (AO (Arbeitsgemeinschaft für Osteosynthesefragen) type 41B or C) of the tibial plateau between 2002 and 2013 at least four years out from surgery were included. Exclusion criteria were extraarticular fractures of the proximal tibia (type AO 41 A) and follow-up of less than four years.

**Figure 1:**
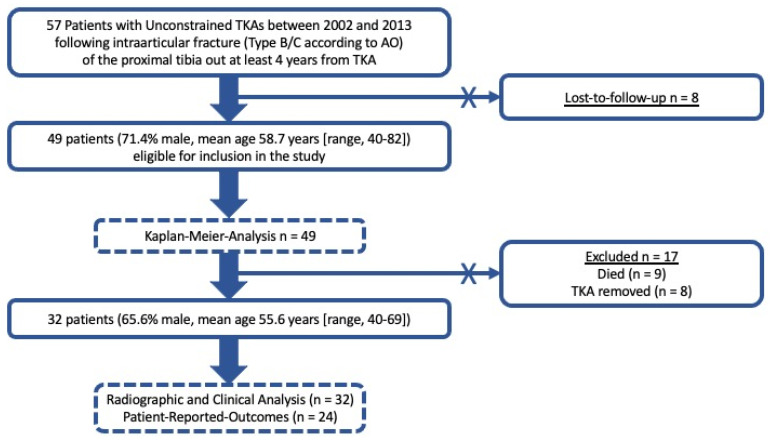
Flow chart of the patient cohort. AO: Arbeitsgemeinschaft für Osteosynthesefragen. TKA: total knee arthroplasty.

A total of 57 patients met the inclusion criteria. Of those, eight patients were lost to follow-up despite multiple attempts to establish contact. This left 49 patients (71.4% male) with a mean age of 58.7 years (range 40–82 years) at the time of TKA for inclusion (Figure 1).

All 49 patients had PTO of the knee as a consequence of an intraarticular proximal tibia fracture type 41 B or C according to the AO and were treated operatively. All initial fractures were treated operatively with internal fixation. The mean age at injury was 46.5 years (range 17–81 years). The right knee was affected in 24 (49.0%) patients, and four (8.2%) patients had open fractures. The injury mechanisms that led to the tibial plateau fracture were falls (22; 44.9%), traffic accidents (21; 42.9%), bicycle accidents (3; 6.1%) and skiing injuries (2; 4.1%). In one patient, the mechanism was unknown. A total of 38 (77.6%) patients had workers’ compensation claims.

All patients underwent unconstrained, cemented TKA at 9.9 (IQR, 1.1–17.4) years from injury at an age of 58.0 (IQR, 53.0–65.0) years. The CKS^®^ (Continuum Knee System, Stratec Medical, Oberdorf, Switzerland) was implanted in 14 patients (28.6%), the LCS^®^ (Complete Knee System, DePuy Synthes, Warsaw, IN, USA) was implanted in 35 (71.4%) patients and navigation was used in 44 (89.8%) patients. Body Mass Index (BMI) was 27.7 (24.7–30.6) kg/m^2^; 22 (42.9%) patients had high blood pressure, 14 (28.6%) patients abused tobacco and 6 (12.2%) patients had diabetes at the time of TKA.

### 2.1. Operative Technique and Postoperative Rehabilitation

A prerequisite for TKA was a prior removal of hardware with microbiological exams and/or joint aspirate indicating a sterile joint before TKA. TKA was performed by or under the supervision of a consultant surgeon with extensive experience in arthroplasty. The operation was carried out under spinal or general anesthesia. Single-shot broad-spectrum antibiotics were administered prior to incision. The standard approach was an anterior midline incision with medial parapatellar arthrotomy. If scars from previous procedures (e.g., osteosynthesis of the tibial plateau fracture) were present and the distance to the anterior midline incision was ≤5 cm, the scar was incorporated into the new incision in order to avoid necrosis of the skin [21]. Mechanical alignment of the knee was aimed for using measured resection starting with the tibia. Prostheses were implanted according to the manufacturers’ instructions. Resurfacing of the patella was not routinely performed. All prostheses were cemented whilst having a tourniquet inflated, and in 44 (89.8%) patients, navigation (OrthoPilot^®^, Braun, Melsungen, Germany) was used. After thorough irrigation, the wound was closed in a layered fashion. Patients were allowed full weight-bearing and unrestricted ROM at postoperative day one. If a drain was placed intraoperatively, it was removed on postoperative day two. Patients received subcutaneous injections of low-molecular-weight heparin (40 mg Clexane^®^, Sanofi, Paris, France) as a thrombosis prophylaxis if not indicated otherwise.

### 2.2. Complications, Revisions and Survival Analysis

Complications and revisions following TKA were recorded. For survival analysis, failure was defined as TKA removal or exchange to revision arthroplasty.

### 2.3. Radiographic and Clinical Evaluation

Patients who did not fail underwent clinical and radiographic evaluation. Preoperatively and at final follow-up, knee range of motion (ROM) was recorded in person with the use of a goniometer. Patient-reported outcome scores (PROs) that were collected included the Oxford Knee Score (OKS) [22] and the Knee Society Score (KSS) [23] at pre- and postoperative levels. Clinical significance measures that have been established for the use of the KSS in TKA were assessed [24,25]. The minimal clinically important difference (MCID) is the minimal change in a score that is experienced by the patient. The MCID has been demonstrated to be 9 points for KSS-Knee and 10 points for KSS-Function [24]. The substantial clinical benefit (SCB) is a measure for the improvement that a patient experiences as clinically considerable. The SCB has been demonstrated to be 40 points for KSS-Knee and 39 points for KSS-Function [24]. The patient-acceptable symptom state (PASS) is a threshold for PROs beyond which patients find themselves to have reached an acceptable outcome. The PASS has been demonstrated to be 85.5 points for KSS-Knee and 77.5 for KSS-Function [25].

Additionally, health-related quality of life (HRQL) was assessed using the Short Form-12 (SF-12) Physical (PCS) and Mental Component Summary (MCS) at final follow-up [26]. Long-leg standing anteroposterior and lateral radiographs were performed pre- and postoperatively. Radiographs were assessed for the anatomical femorotibial angle (aFTA) and proximal tibial slope (PTS).

### 2.4. Statistical Analysis

Normality of data was assessed using the Shapiro–Wilk test. Normally distributed, continuous data were reported as mean and range, and skewed, continuous data as median with interquartile range (IQR). Parametric, continuous data were analyzed using the independent samples or paired *t*-test, and nonparametric data were reported using the Mann–Whitney U test or Wilcoxon’s matched pairs test. Fisher’s exact test was used to analyze bivariate or categorical data. Subgroup analyses were performed for the type of implant used (LCS vs. CKS), age (≥60 vs. <60 years) and sex (male vs. female). To assess for statistical significance, two-tailed *p* values were calculated, and significance was set at *p* < 0.05. Kaplan–Meier survival analysis was performed, and a life table was used to examine and depict TKA survival. TKA failure was defined as exchange or removal of the prosthesis. Statistical analysis was performed by one of the authors (PCN) using Prism software (GraphPad, version 10.0.2, San Diego, CA, USA).

## 3. Results

### 3.1. Complications, Revisions and Survival Analysis

A total of 15 (30.6%) patients had intra- and/or postoperative complications. The patellar tendon was intraoperatively injured and fixed in two (4.1%) patients. One of those patients had a subsequent periprosthetic joint infection (PPJI) and ultimately underwent TKA removal.

A total of 14 (28.6%) patients had a postoperative complication. The most common complications were PPJIs (7 patients; 14.3%), followed by stiffness (range of motion ≤ 90 degrees (4 patients; 8.2%)), instability, aseptic loosening and postoperative leukocytosis/fever necessitating arthroscopic irrigation (all 1 patient; 2.0%).

Revision surgery was performed in 10 (20.4%) patients. Of those, two (4.1%) patients had an early PPJI and underwent irrigation and debridement with prosthesis retention. The remaining eight (16.3%) patients underwent prosthesis removal due to PPJI (six patients; 12.2%), instability or aseptic loosening (both one patient; 2.0%) at median 2.5 years (IQR, 0.5–5.8 years).

In the six patients with PPJI who had their prosthesis removed, microbiological results demonstrated coagulase-negative staphylococci (four patients, 8.2%) and streptococcus pneumoniae (one patient; 2.0%). In one (2.0%) patient, no pathogen could be isolated, but joint aspirate, blood work (white cell blood count, CRP) and intraoperative findings were consistent with PPJI.

**Figure 2:**
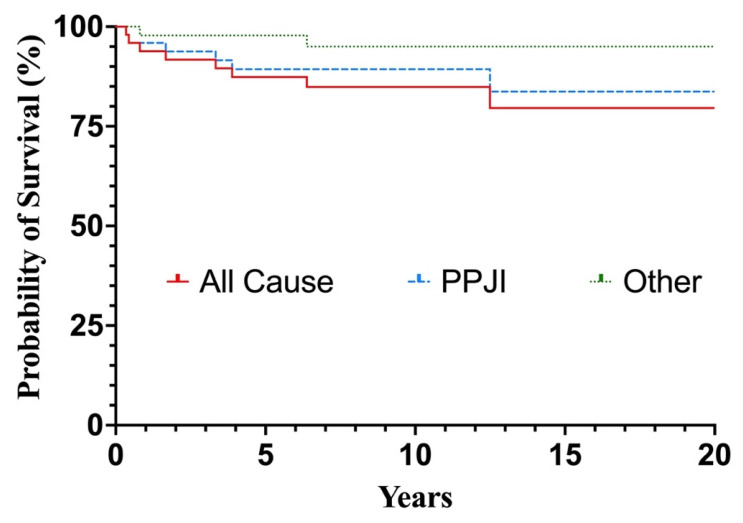
Kaplan–Meier curve demonstrating the probability of total knee arthroplasty survival. PPJI: periprosthetic joint infection.

Kaplan–Meier survival analysis demonstrated a cumulative survival rate of 79.6% at 20 years with removal of the TKA for any cause defined as failure. Survival analysis displayed a relatively steep decline in the first five years, with a gradual subsequent decline then reaching a plateau at 12.5 years (Figure 2). The highest annual failure rate was observed in the first five years, in which six patients (12.4%) underwent implant removal (Table 1).

No significant differences were found for the type of implant used. There were three (6.1%) patients that had their CKS^®^ prosthesis removed and five (10.2%) patients that had their LCS^®^ prosthesis removed (*p* = 0.673). Similarly, there were no significant differences when failure rates for male (six; 12.2%) and female (two; 4.1% (*p* > 0.999)) patients as well as patients below the age of 60 years (three; 6.1%) and patients 60 years or older (five; 10.2% (*p* = 0.245) at the time of TKA were compared.

### 3.2. Clinical and Radiographic Results

Following exclusion of the patients who died (9 patients; 18.4%) or underwent implant removal or exchange (8 patients; 16.3%), a total of 32 patients (65.6% male, mean age 55.6 years (range, 40–69)) were available for further analysis. The mean follow-up was 11.8 years (95% CI, 10.4–13.3 years). Range of motion (*p =* 0.028), extension (*p* < 0.001), aFTA (*p* = 0.044) and pPS (*p* = 0.009) all improved significantly over preoperative levels. There was a slight increase in flexion; however, this was not significant (*p* = 0.196). Clinical and radiographic outcomes are presented in Table 2.

Complete pre- and postoperative PROs (OKS and KSS) and postoperative HRQL assessment (SF-12) were available for 22 patients (Table 3). Both OKS (*p* < 0.001) and KSS (*p* < 0.001) significantly improved over preoperative levels. The MCID for KSS-Knee and KSS-Function was achieved in 19 (86.4%) and 18 (81.8%) patients. The SCB for the KSS-Knee and KSS-Function was achieved in 10 (45.5%) and 10 (45.5%) patients, respectively. The PASS for the KSS-Knee and KSS-Function was reached in 6 (27.3%) and 15 (68.2%) patients.

## 4. Discussion

The most important finding of this study was that total knee arthroplasty for posttraumatic osteoarthritis because of intraarticular fractures of the tibial plateau has a high rate of complications (30.6%), revision surgery (20.4%) and implant removal (16.4%), resulting in an implant survival rate of 84.9% at 10 years and 79.6% at 20 years as shown in the Kaplan–Meier survival analysis. Survival analysis demonstrated a relatively steep decline in the first five years, with a gradual subsequent decline reaching a plateau at 12.5 years. The reasons for failure—defined as implant removal due to any cause—were periprosthetic joint infections (12.2%), instability and aseptic loosening (both 2.0%).

The present findings are corroborated by Houdek et al. [15] who compared TKA for patients with PTO (n = 531) to that for patients with primary OA (n = 19,641) at a mean follow-up of 6 years. However, of 521 patients with PTO, only 341 patients had a previous proximal tibia fracture with no further information on the percentage of intraarticular fractures. A total of 56 patients (11%) underwent revision TKA at a mean of 4 years following primary TKA, and the most common indication for revision TKA was infection (n = 22; 4%). Both the timing and the indication for revision TKA are similar to our findings. The 10- and 20-year survival rates were 88% and 67% for TKA in patients with PTO compared to 96% and 75% for patients with OA (*p* < 0.0001). Although the authors included both femoral and tibial fractures, they did not find a significant difference in survival rates between the two. However, in contrast to the present study, a significantly higher risk for revision TKA was demonstrated for patients older than 60 years (*p* < 0.0001).

Scott et al. [13] investigated 31 patients with PTO who were treated with TKA. This study is comparable to the present work in terms of design, as all patients had intraarticular tibial plateau fractures. Of 31 patients, 24 had previously undergone open reduction and internal fixation. The authors demonstrated an intraoperative complication rate of 10%, a late complication rate of 22% and all-cause survival of 82.3% at ten years. Interestingly, four (12.8%) patients with a superficial wound infection/dehiscence did not require surgical intervention but settled with antibiotics, and only one (3.2%) patient underwent revision for a periprosthetic (deep) joint infection.

Lizaur-Utrilla et al. [27] performed a prospective study comparing TKA for primary OA vs. TKA for PTO following tibial plateau fracture. The authors included 29 patients with TKA for PTO and demonstrated a 13.7% complication rate with no PPJIs and only one patient requiring implant exchange due to mechanical failure at 6 years.

Lunebourg et al. [28] performed a matched cohort study and demonstrated a significant difference in the 10-year survival rate (*p* < 0.001) between 33 patients with PTO and 407 patients with OA. With failure defined as “any surgery on the operated knee”, the survival rate in the OA group was 99%, whereas for patients with PTO, survival was only at 79%. Additionally, the authors found a significantly higher overall complication rate for PTO (21.2%) compared to that for OA (1.2%; *p* < 0.001). Compared to the present study (14.3%), Lunebourg et al. [28] reported fewer cases of deep infection (6.1%). Among the reasons for this difference may be that patients with PTO due to fractures of both the tibia and the femur were included. Furthermore, one third of patients only had extraarticular fractures, potentially highlighting a lesser injury severity.

Putman et al. [19] investigated the 10-year survival rate of TKA in patients with PTO and found a survival rate of 89% with the most common complication being infection (4.5%). Of note, patients were included if they had PTO from either fracture, ligament injury or osteotomies, making this a relatively heterogeneous cohort. Out of 263 TKAs, only 32 were intraarticular fractures involving the tibial plateau.

In a recent systematic review, Syrikas et al. [11] demonstrated a higher postoperative complication rate with infections being the most common, a higher revision rate and inferior patient-reported outcomes in patients with TKA following PTO when compared to those of OA.

Despite the high complication and revision rate our data demonstrates that TKA for PTO following intraarticular tibial plateau fractures provides significant improvement in PROs and knee ROM. Specifically, a preoperative knee extension deficit of five degrees was corrected to zero degrees, which is an important prerequisite for physiological gait. This was also shown by Lizaur-Utrilla et al. [27], Putman et al. [19] and Lunebourg et al. [28], all of whom demonstrated significant improvements in range of motion. In the present cohort, aFTA and pPS were significantly improved from 170 to 173 degrees and 8 to 6 degrees at final follow-up. Similar postoperative values were demonstrated by Scott et al. [13], who published an aFTA of mean 174 degrees in patients after TKA as a result of tibial plateau fractures. Among the reasons for TKA failure is poor component alignment. This includes varus and valgus malalignment, but also malrotation [29,30]. This was not further investigated in this study but should be in future research.

Lunebourg et al. [28] demonstrated significantly better values for KSS Knee and KSS Function in patients who underwent TKA for primary OA compared to those who underwent PTO. The values demonstrated for TKA after PTO closely resemble our data (KSS Knee 77 vs. 76.5, KSS Function 81 vs. 80.0). This finding is reassuring, as Lunebourg et al. likely had a lower injury severity as one third of patients in their cohort had extraarticular fractures. On the other hand, Lizaur-Utrilla et al. [27] found no significant differences in KSS between TKA for patients with OA vs. PTO. However, the postoperative values for the PTO group were better compared to those in our data (KSS Knee 86.2 vs. 76.5, KSS Function 87.9 vs. 80.0). In contrast to Scott et al. [13], we found a significant improvement in OKS from pre- to postoperative values for our patients. Interestingly, when comparing the postoperative values of the SF-12, our values are closer to what Scott et al. found for TKA following primary OA. Similar values for the SF-12 were demonstrated by Lizaur-Utrilla et al. [27] and Khoshbin et al. [31].

In this study, we investigated clinical significance measures (e.g., MCID, SCB, PASS) by calculating how many patients reached the thresholds described in the literature [24,25]. We found that, although significant improvements in the KSS from pre- to postoperative conditions were demonstrated, only 80% reached the MCID, and fewer than 50% of patients reached the threshold for SCB. Further, the number of patients reaching the PASS was quite heterogeneous, with 27% for KSS Knee and 68% for KSS Function. In other words, one in five patients following TKA for PTO did not experience a clinically meaningful difference in the outcome, and only half of patients experienced a substantial clinical benefit from the procedure. It is important to note that the thresholds for MCID, SCB and PASS that were used in this study were established for patients following TKA for primary OA and not for patients who underwent TKA for PTO.

### Limitations

The present study is not without limitations. First, this is a single-center, retrospective study, and it is therefore prone to bias and loss of data. Second, the sample size is relatively small, although this is in part due to the rarity of TKA in PTO. Also, there was no control group. However, this study reports one of the largest cohorts with one of the longest follow-ups that has been reported to date.

## 5. Conclusions

One third of patients suffer a complication and one in six patients has their prosthesis removed after total knee arthroplasty for posttraumatic osteoarthritis due to tibial plateau fractures amounting to a cumulative survival rate of 79.6% at 20 years. In patients who do not fail, total knee arthroplasty significantly improves clinical and radiographic outcomes at long-term follow-up. 

## Figures and Tables

**Table 1:** Life Table.

Time after TKA	Implant Survival (%)	95% CI
1 year	93.8	89.7–100
5 years	87.4	80.7–100
10 years	84.9	77.3–99.0
20 years	79.6	69.2–98.1

CI: confidence interval. TKA: total knee arthroplasty.

**Table 2:** Clinical and radiographic variables at pre- and postoperative levels.

Clinical and Radiographic Variables	Preoperative	Postoperative	*p*-Value
Range of Motion, in °, mean (95% CI)	99 (92–105)	106 (101–111)	0.028
Extension, in °, median (IQR)	5 (0–10)	0 (0–0)	<0.001
Flexion, in °, mean (95% CI)	104 (98–109)	108 (102–113)	0.195
aFTA, in °, mean (95% CI)	170 (168–173)	173 (172–174)	0.044
pPS, in °, mean (95% CI)	8 (7–10)	6 (6–7)	0.009

aFTA: anatomical femorotibial angle, CI: confidence interval, pPS: proximal posterior slope. IQR: interquartile range.

**Table 3:** Patient-reported outcomes and health-related quality of life assessment at pre- and postoperative levels.

PROs and HRQL Assessment	Preoperative	Postoperative	*p*-Value
OKS, mean (95% CI)	20.7 (15.9–25.5)	37.7 (34.6–40.8)	<0.001
KSS total, mean (95% CI)	82.8 (66.1–99.5)	155.5 (143.8–167.1)	<0.001
Knee, median (IQR)	36.5 (17.3–51.0)	76.5 (68.8–89.0)	<0.001
Functional, median (IQR)	50.0 (40.0–60.0)	80.0 (67.5–100)	<0.001
SF-12 PCS SF-12 MCS	n.a. n.a.	42.3 (37.7–47.0) 54.4 (51.0–57.8)	n.a. n.a.

CI: confidence interval, HQRL: and health-related quality of life, KSS: Knee Society Score, OKS: Oxford Knee Score, MCS: mental component summary, PROs: Patient-reported outcomes, PCS: physical component summary, SF-12: Short Form-12.

## Data Availability

All data and materials regarding the study are available from the corresponding author on reasonable request.
